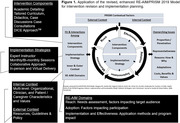# Qualitative use of the RE‐AIM/PRISM framework for adaptation of a primary care educational program on the care of persons with behavioral and psychological symptoms of dementia

**DOI:** 10.1002/alz.095321

**Published:** 2025-01-09

**Authors:** Tamara J. LeCaire, Molly Schroeder, Uriel Paniagua, Jonathan A Stone, Tammi Albrecht, Stephanie Houston, Sarina Schrager, Cynthia M. Carlsson, Art Walaszek

**Affiliations:** ^1^ Wisconsin Alzheimer’s Institute, University of Wisconsin School of Medicine and Public Health, Madison, WI USA; ^2^ Wisconsin Alzheimer's Institute, University of Wisconsin School of Medicine and Public Health, Madison, WI USA; ^3^ Oakland University William Beaumont School of Medicine, Rochester, MI USA; ^4^ Wisconsin Research and Education Network, University of Wisconsin School of Medicine and Public Health, Madison, WI USA; ^5^ Wisconsin Alzheimer’s Disease Research Center, University of Wisconsin School of Medicine and Public Health, Madison, WI USA

## Abstract

**Background:**

Primary care clinicians report limited self‐efficacy for managing complex behavioral and psychological symptoms in persons with dementia (BPSD). Complementary approaches, the academic detailing model and the DICE Approach^TM^, improved self‐efficacy for memory clinic clinicians and their teams to identify and manage BPSD. Methods guided by the RE‐AIM (Reach, Effectiveness, Adoption, Implementation, Maintenance) / PRISM (Practical, Robust, Implementation and Sustainability Model) framework are shared for pre‐implementation adaptation of this program for generalist primary care.

**Methods:**

A stakeholder‐engaged qualitative study with both current and future program stakeholders was used to identify a) gaps in BPSD management, and b) multi‐level barriers and facilitators to the academic detailing model. Framework guided semi‐structured interview topics included contextual factors impacting program reach, reasons for adoption and program effectiveness, and barriers and facilitators for implementation. The team completed 14 interviews; 7 with clinicians practicing in primary care integrated memory clinics with prior academic detailing participation (Champions) and among 7 clinician colleagues with no or minimal program experience (Novices). Inductive and deductive qualitative thematic analysis approaches were used, the latter organized by RE‐AIM domains and multi‐level context.

**Results:**

A sample of themes and adaptations for shaping program access and fit (Reach) are shared. 1) Applied learning supports knowledge and self‐efficacy for BPSD care. Case‐based, experiential learning fits for both experienced and inexperienced clinicians. Champions acknowledge experiential learning builds confidence (facilitator), while Novices note having limited direct care experience (barrier). Adaptations for Novices will include more extensive introduction to identifying and managing BPSD and emphasize building confidence. 2) Improve access to education and materials. Champions noted the challenge to “keep tools at your fingertips” could be overcome with reference materials or templates while Novices highlighted needing access to more structured resources in light of numerous guidelines to follow in primary care. In response, user‐friendly BPSD management resource guides to support clinical decisions have been developed.

**Conclusions:**

We provide a novel qualitative application RE‐AIM/PRISM with both current and future stakeholders to inform adaptations pre‐implementation. This approach will aid identifying contextual barriers and facilitators to reach, adoption, implementation, and effectiveness of academic detailing for delivery to generalist primary care teams.